# Comparison of the effects of transperitoneal and retroperitoneal robot-assisted partial nephrectomy

**DOI:** 10.12669/pjms.40.10.10613

**Published:** 2024-11

**Authors:** Meiman Tao, Kang Cheng, Wei Xu, Zhounan Qian, Peng Pan

**Affiliations:** 1 Meiman Tao, Department of Urology, Affiliated Hospital of Jiangsu University, Zhenjiang City, Jiangsu Province, 212001, China; 2 Kang Cheng, Department of Urology, Affiliated Hospital of Jiangsu University, Zhenjiang City, Jiangsu Province, 212001, China; 3 Wei Xu Department of General Surgery, The Fourth People’s Hospital of Zhenjiang, Zhenjiang City, Jiangsu Province, 212000, China; 4 Zhounan Qian, Department of Urology, Affiliated Hospital of Jiangsu University, Zhenjiang City, Jiangsu Province, 212001, China; 5 Peng Pan, Department of Urology, Affiliated Hospital of Jiangsu University, Zhenjiang City, Jiangsu Province, 212001, China

**Keywords:** Robot-assisted partial nephrectomy, Renal cell carcinoma, Retroperitoneal, Transperitoneal

## Abstract

**Objective:**

To compare the effects of transperitoneal and retroperitoneal approaches for robotic assisted partial nephrectomy (RAPN) in patients with renal cell carcinoma (RCC).

**Methods:**

We conducted a retrospective cohort study on RAPN at Affiliated Hospital of Jiangsu University. Between September 2020 and February 2024, the included patients underwent either transperitoneal approach or retroperitoneal approach. Perioperative status, stress response, quality of life, and incidence of complications were compared between the groups.

**Results:**

A total of 105 patients were included in this analysis, with 54 patients in the Retroperitoneal group, and 51 patients in the Transperitoneal group. The retroperitoneal approach was associated with significantly better perioperative indicators compared to the transperitoneal method (*P*<0.05). After the surgery, serum levels of interleukin-6 (IL-6), C-reactive protein (CRP), white blood cell count (WBC), and cortisol (Cor) in the Retroperitoneal group were lower than in the Transperitoneal group (*P*<0.05). The quality-of-life scores of patients in the Retroperitoneal group were higher (*P*<0.05), but no statistically significant difference in the incidence of complications between the groups (*P*>0.05).

**Conclusions:**

Compared with the transperitoneal approach, the retroperitoneal method of RAPN is equally safe and is associated with improved perioperative status, lower stress response, and better quality of life for RCC patients.

## INTRODUCTION

Renal cell carcinoma (RCC) originates from the renal epithelium, and accounts for over 90% of all kidney cancers.^1^,^2^ Surgical intervention is the preferred treatment for early and mid-stage RCC and is associated with good clinical outcomes and no need for subsequent chemotherapy or radiation therapy.^3^-^5^ However, the effects of different surgical approaches and of zero ischaemia on the postoperative reduction in renal function remain controversial. We aimed to investigate the relative short- and long-term changes in estimated glomerular filtration rate (eGFR However, traditional open surgery is accompanied by significant trauma.^6^,^7^ Recently, laparoscopic and robot-assisted laparoscopic minimally invasive surgeries have gained popularity due to minimal trauma and a good prognosis. Studies show that robotic-assisted partial nephrectomy (RAPN) can reduce the trauma caused by invasive surgery and improve the effectiveness and safety of treatment by using anatomical structures as markers for surgical treatment.^4^-^8^

Currently, RAPN can be done with either a transperitoneal or retroperitoneal approach. While the transperitoneal approach allows for increased manipulating space for the devices, the retroperitoneal approach is performed in smaller retroperitoneal space and, therefore, requires more extensive training. However, this approach is associated with lower gastrointestinal complications and urine leaks outside of the peritoneum.^5^-^9^ Currently, there is still no consensus on which is the most efficient RAPN approach. These two approaches have similar postoperative, functional, and oncological outcomes for patients with posterolateral renal tumors, although the retroperitoneal approach has shorter operative time. However, a meta-analysis by Zhou J et al.^10^ and a most recent meta-analysis by Shrivastava N^11^ reported that the retroperitoneal approach is superior to the transperitoneal approach in terms of operative time, estimated blood loss, and postoperative length of hospital stay. Even so, Zhou’s report did not show any significant differences in perioperative complications while Shrivastava’s report showed a significantly lower complication rate for the retroperitoneal approach. This study aimed to retrospectively analyze the clinical data of RCC patients of the Affiliated Hospital of Jiangsu University to clarify the application effects of different RAPN approaches in patients with RCC.

## METHODS

Clinical records of patients who underwent RAPN at the Affiliated Hospital of Jiangsu University from September 2020 to February 2024 were retrospectively analyzed in this retrospective study. According to the different surgical approaches, patients were stratified into two groups: Transperitoneal group and Retroperitoneal group.

### Inclusion & Exclusion Criteria:

Patients diagnosed with unilateral RCC^12^ and undergoing RAPN surgery were included. Patients with history of drug and alcohol dependence, abnormal coagulation function, systemic infectious diseases, cardiovascular and cerebrovascular diseases, blood system diseases, or incomplete data were excluded. All procedures performed in this study involving human participants were in accordance with the ethical standards of the institutional and/or national research committee, and the 1964 Declaration of Helsinki and its later amendments or comparable ethical standards.

### Ethical Approval:

This study was approved by the Ethics Review Board of Affiliated Hospital of Jiangsu University (No. LZ2024046; April 23, 2024).

### Surgical procedures:

The procedures were performed by the same group of experienced surgeons.

### (1)Retroperitoneal approach:

The procedure was performed using the da Vinci surgical robot system. Computed tomography (CT) and arterial CT-enhanced angiography were performed before the surgery, and the anatomical location and volume of the tumor and blood vessels were determined through three-dimensional reconstruction. Under general anesthesia, the lumbar bridge was elevated appropriately, and the patient was placed on the healthy side. Retroperitoneal space was dilated, with the first puncture point located approximately 2cm below the rib margin of the midaxillary line. Blunt separation of the subcutaneous muscle layer and lumbar fascia layer was performed using vascular forceps.

The peritoneum was separated using the index finger. For overweight patients, an airbag was placed in the retroperitoneal space, and 600 ml of gas was injected. After about five minutes, the gas inside the airbag was discharged. For patients with normal weight, the index finger was inserted and used as a guide to make the second puncture point on the skin about 1cm below the axillary line. The third puncture point was made 2cm above the iliac crest. Trocar was inserted, and a 10mm incision was made in the midaxillary line. After inserting a cannula, suturing was performed. Gas was injected and the pressure value was maintained at 15 mmHg. An observation mirror was inserted into the casing. Assisted by laparoscopy, the surgical procedure was performed to clarify the peritoneal fold and lumbar muscle anatomy upon reaching the abdominal cavity.

The perirenal fascia was opened near the peritoneal fold, and ureteral dissociation was performed at a distance of 2cm from the upper/lower pole of the kidney. The kidney was released along the psoas major muscle. After clarifying the location of the renal artery, the surrounding tissues were separated layer by layer, and the ventral renal pedicle was freed. If the operating area was narrow, the kidney was gently rotated and freed to fully expose the diseased tissue. Renal blood vessels were clumped, and the diseased tissue was removed together with the 0.5-1 cm margin. A catheter was placed for drainage, the incision was closed, and the remaining fat on the surface of the kidney was fixed to the surrounding tissue using a Hem-o-lok clamp, restoring the anatomical position of the kidney.

### Transperitoneal approach:

Patients were placed in a healthy lateral position, and a 1cm incision was made next to the navel. A pneumoperitoneum needle was inserted, an artificial pneumoperitoneum was constructed and placed by laparoscopy. The laparoscopic-assisted incision was made at the level of the iliac crest and umbilicus, as well as approximately 2cm below the outer rib margin of the rectus abdominis muscle, and a trocar was inserted. The incision raised and lowered the para colon sulcus, and the colon and mesentery were released downwards. The remaining procedures were similar to the retroperitoneal approach.

### Observation indicators:

- Perioperative conditions, including surgical duration, intraoperative blood loss, postoperative ventilation duration, start of food intake, and length of hospital stay.
- Stress response indicators, including levels of interleukin-6 (IL-6), C-reactive protein (CRP), white blood cell count (WBC), and cortisol (Cor), were measured in the 2ml of fasting venous blood by enzyme-linked immunosorbent assay.
- Quality of life: The European Organization for Research and Treatment of Cancer Quality of Life Questionnaire Core 30 (EORTC QLQ-C30) was used to evaluate cognitive, social, emotional, and physical functions.^13^ The score range for each dimension was 0-100 points, and the higher the score, the better the quality of life.
- Complications, including renal dysfunction, hematuria, subcutaneous edema, and infection.

### Statistical Analysis:

All data analyses were conducted according to predefined statistical analysis plans using SPSS version 25.0 software (IBM, Armonk, New York, USA). For continuous variables, the means and standard deviations (SD) were calculated. The independent sample *t*-test was used to compare the mean of two independent samples, especially for continuous variables. The hypothesis of equal variance was examined and considered in the analysis. For categorical variables, frequency distribution was provided and expressed as a percentage. The chi square test was used to compare categorical variables, such as gender distribution and tumor location, between two groups. A *p*-value less than 0.05 was considered statistically significant.

## RESULTS

**Fig.1:**
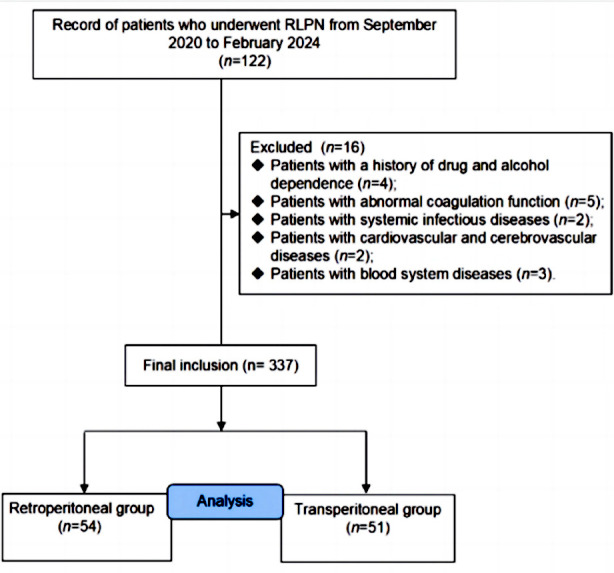
Guidelines Flow Diagram.

Clinical records of 122 patients with RAPN were considered for this retrospective study. Of them, 105 patients (71 males and 34 females) met eligibility criteria. There were 54 cases in the Retroperitoneal group and 51 cases in the Transperitoneal group (Fig.1), with no statistically significant difference in general information between the two groups of patients (*P*>0.05) (Table-I).

**Table-I:** Comparison of General Information between Two Groups.

Item	Retroperitoneal group (n=54)	Transperitoneal roup (n=51)	t/χ^2^	P
***Gender [n (%)]***				
Male	35 (64.81)	36 (70.59)	0.399	0.527
Female	19 (35.19)	15 (29.41)		
Age (year)	50.15±6.38	48.92±7.91	0.879	0.381
Lesion diameter (cm)	3.78±0.91	3.92±0.86	0.809	0.420
***Affected side [n (%)]***				
Left	29 (53.70)	25 (49.02)	0.230	0.631
Right	25 (46.30)	26 (50.98)		
BMI (kg/m^2^)	23.47±2.59	23.73±2.39	0.534	0.595
***Tumor location [n (%)]***				
Upper pole	37 (68.52)	36 (70.59)	0.054	0.974
Middle pole	9 (16.67)	8 (15.69)		
Lower pole	8 (14.81)	7 (13.73)		

**Fig.2:**
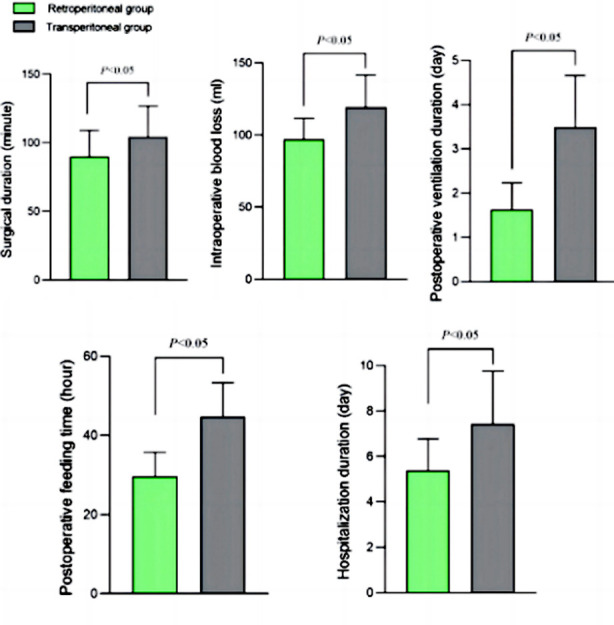
Comparison of perioperative conditions between two groups.

The duration of surgery, postoperative ventilation, start of ingestion, and length of hospital stay in the Retroperitoneal group were shorter, and the intraoperative blood loss was lower than in the Transperitoneal group (*P*<0.05) (Fig.2).

**Fig.3:**
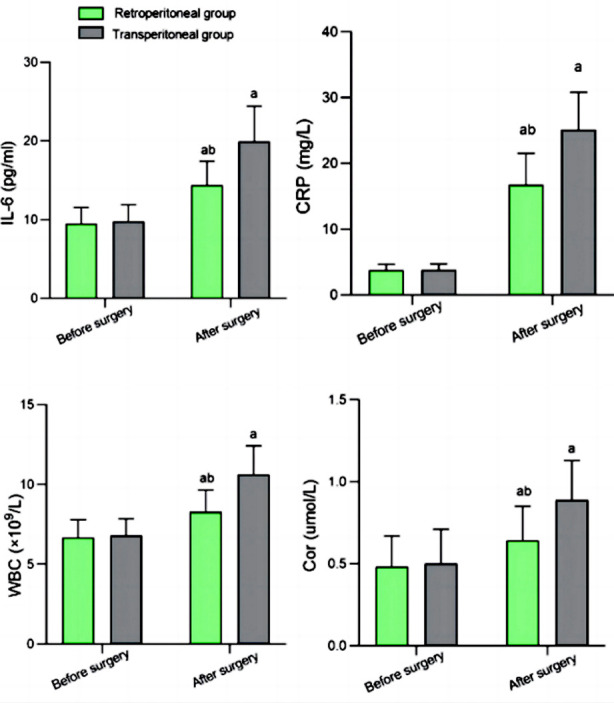
Comparison of stress response index levels between two groups; Compared with the same group before surgery, aP<0.05; Compared to Transperitoneal group, bP<0.05; IL-6: interleukin-6; CRP: C-reactive protein; WBC: white blood cell count; Cor: cortisol.

Before the surgery, there was no significant difference in serum levels of IL-6, CRP, WBC, and Cor in both groups (*P*>0.05). After surgery, serum levels of IL-6, CRP, WBC, and Cor in both groups increased but were significantly lower in the Retroperitoneal group compared to the Transperitoneal group (*P*<0.05) (Fig.3).

**Fig.4:**
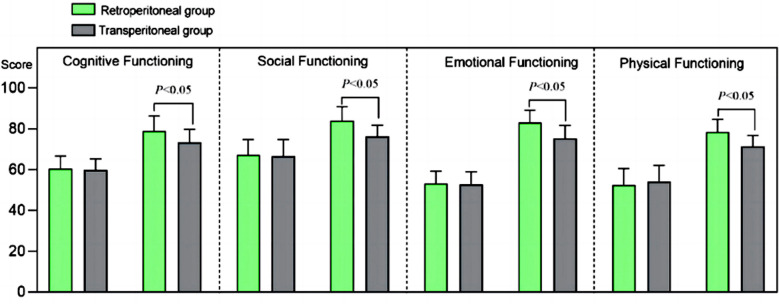
Comparison of Quality of Life between Two Groups.

Pre-surgery EORTC QLQ-C30 scores of cognitive, social, emotional, and physical functioning were comparable in both groups (*P*>0.05). After the surgery, all EORTC QLQ-C30 scores in both groups increased and were considerably higher in the Retroperitoneal group compared to the Transperitoneal group (*P*<0.05) (Fig.4). There was no statistically significant difference in the incidence rate of complications between the two groups (*P*>0.05) (Table-II).

**Table-II:** Comparison of incidence rates of complications between two groups.

Group	n	Abnormal renal function	Hematuria	Subcutaneous edema	Infect	Total incidence rate
Retroperitoneal group	54	0 (0.00)	1 (1.85)	1 (1.85)	0 (0.00)	2 (3.70)
Transperitoneal group	51	2 (3.92)	2 (3.92)	3 (5.88)	1 (1.96)	8 (15.69)
*χ^2^*						3.090
*P*						0.079

## DISCUSSION

This study retrospectively analyzed the clinical data of 105 RCC patients who underwent RAPN. The results showed that the retroperitoneal RAPN approach was associated with lower surgical duration and postoperative ventilation time, quicker start of feeding, shorter hospital stay, and lower intraoperative blood loss compared to the transperitoneal approach. The retroperitoneal RAPN was also linked to lower stress response and considerably better quality of life than the transperitoneal approach.

Both retroperitoneal and transperitoneal RAPN are important clinical treatments for RCC.^11^,^14^ Similar with Shrivastava N^11^, a study by Liao XH et al.^14^ that included RCC patients with ≥ T1b stage found that retroperitoneal laparoscopic total renal artery occlusion can effectively protect renal function without increasing intraoperative blood loss and the risk of postoperative complications. A propensity score-matched comparative analysis by Takagi T et al.^15^ also demonstrated that compared to the transperitoneal approach, the retroperitoneal method has significant advantages in terms of surgical time, estimated blood loss and postoperative length of hospital stay. Lanzotti NJ et al.^16^ investigated the difference in the application effect of retroperitoneal and retroperitoneal approaches for radical nephrectomy in RCC patients and also confirmed that the retroperitoneal approach could reduce surgical trauma and lower the risk of complications. In contrast, the study by Tang H^17^ showed that in RCC patients, both transperitoneal and retroperitoneal approaches were associated with similar surgical time, renal artery occlusion time, intraoperative blood loss, positive surgical margin rate, and incidence of postoperative complications. Their study only reported shorter postoperative recovery time for intestinal function in patients who underwent retroperitoneal RAPN compared to transperitoneal surgery.^17^ The results of this present study are generally consistent with those previous studies by Shrivastava N^11^, Liao XH et al.^14^, Takagi T et al.^15^, and Lanzotti NJ et al.^16^, except with regard to incidence rate of complications. This discrepancy may be related to the different populations, disease stages, severity of the selected cases, and sample size.

The transperitoneal approach requires inserting the instruments into the abdominal cavity, which has the advantages of an obvious anatomical position and large operating space. Therefore, this approach is convenient for the treatment of large-volume tumors and makes it easier to address injuries that may occur during the surgery.^10^,^18^,^19^ However, during the surgery, it is necessary to separate the peritoneum and organize the intestinal mesentery, which can easily lead to complications such as intestinal obstruction and paralysis. Moreover, exposing tumor tissue takes a long time, which may negatively impact postoperative eating and recovery times.^18^,^19^

The retroperitoneal approach, which involves mechanical insertion into the abdominal cavity through the retroperitoneum, allows to obtain a faster surgical field of view and requires less free peritoneum and mesentery.^19^,^20^ Therefore, this method is associated with shorter surgical time, as well as time for postoperative food intake and faster recovery. However, retroperitoneal access results in a narrower surgical field of view, which makes identifying the anatomical landmarks more difficult and, therefore, requires extensive training.^20^-^22^

Our results also reported that the retroperitoneal approach was associated with a significantly lower stress response, as indicated by lower serum levels of IL-6, CRP, WBC, and Cor. Furthermore, quality of life scores in patients who underwent surgery with the retroperitoneal approach were higher compared to the Transperitoneal group, while the incidence of complications was comparable. Our results further confirm the feasibility and effectiveness of the retroperitoneal RAPN for RCC.

The advantages of the retroperitoneal approach that were identified in our study and confirmed by previous research are of great clinical significance for improving the effectiveness and safety of RCC treatment. The retroperitoneal approach can reduce interference with abdominal organs, facilitate the separation, exposure, and clamping of renal blood vessels, accurately control renal pedicle blood vessels, reduce surgical trauma, improve surgical efficiency, reduce surgical treatment time, and shorten postoperative rehabilitation time.^23^,^24^

### Limitations:

It is a single-center retrospective analysis with a small sample size. The cohort included in the study was not homogeneous in clinical and pathological diagnosis. The impact of the two methods on the long-term functional recovery of patients was not analyzed. The data of this study need to be confirmed through further research on expanding and specific patient cohorts. Finally, we could not eliminate the potential impact of drugs on the obtained results. Further high-quality research is needed to validate the conclusions of this study.

## CONCLUSION

Compared with the transperitoneal approach, a retroperitoneal RAPN for RCC can reduce trauma, shorten postoperative recovery time, alleviate stress reactions caused by invasive surgical procedures, improve patient quality of life, and with equally safe profile.

### Authors’ contributions:

**MT:** Conceived designed the study and Review. Was involved in preparing the manuscript.

**KC, WX, ZQ and PP:** Collected the data .performed the analysis and Review.

All authors have read and approved the final manuscript and are responsible for the integrity of the study.
